# Spatial patterns of intrinsic brain activity in rats with capsular stroke

**DOI:** 10.1002/brb3.3125

**Published:** 2023-07-06

**Authors:** Jie Ma, Xue‐Jia Li, Wen‐Xin Liu, Fei Teng, Xu‐Yun Hua

**Affiliations:** ^1^ Center of Rehabilitation Medicine Yueyang Hospital of Integrated Traditional Chinese and Western Medicine, Shanghai University of Traditional Chinese Medicine Shanghai China; ^2^ Department of Traumatology and Orthopedics Yueyang Hospital of Integrated Traditional Chinese and Western Medicine, Shanghai University of Traditional Chinese Medicine Shanghai China; ^3^ Emergency Medicine Clinical Research Center, Beijing Chaoyang Hospital Capital Medical University, & Beijing Key Laboratory of Cardiopulmonary Cerebral Resuscitation Beijing China; ^4^ Yangzhi Rehabilitation Hospital (Shanghai Sunshine Rehabilitation Center) Tongji University Shanghai China

**Keywords:** capsular infarct, cortical activity, functional magnetic resonance imaging, neural plasticity, stroke

## Abstract

**Background:**

To explore the neural changes of brain activity in rats with circumscribed capsular infarcts to find a new therapeutic target for promoting the functional recovery.

**Methods:**

A total of 18 capsular infarct rats and 18 normal rats were conducted in this study. All animal use procedures were strictly in accordance with the guide for the care and use of laboratory animals. After establishing the photothrombotic capsular infarct model, the functional magnetic resonance imaging (fMRI) data were collected and analyzed.

**Results:**

The fMRI results indicated that the passive movement would induce strong activation in caudate, putamen, frontal association somatosensory cortex, thalamus dorsolateral, and thalamus midline dorsal in control group, and the passive movement would only induce limited activation mostly in somatosensory cortex, thalamus dorsolateral, and thalamus midline dorsal in capsular infarct models. Capsular infarct makes the cortical activity weaken in sensory‐related cortex and subcortical nuclei, including capsular area and thalamus.

**Conclusions:**

Such findings imply that the posterior limb of internal capsule (PLIC) is connected to these structures in function, interacts together with them, and, accordingly, the lesion of PLIC manifests the related symptoms.

## Graphical abstract

The findings imply that the posterior limb of internal capsule is connected to these structures in function, interacts together with them, and, accordingly, the lesion of the posterior limb of internal capsule manifests the related symptoms.

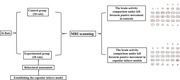

## 1 INTRODUCTION

Subcortical infarcts account for nearly a quarter of strokes, mostly found at the site of terminal–arterial of the white matters (Bamford et al., 1991). The annual mortality of stroke decreases in recent years, whereas the incidence of stroke affecting the subcortical white matter is increasing in the elders, which demands an intensive study in this field (Roger et al., 2012). Despite the urgent requests, fewer longitudinal studies have explored the changes in brain activity after subcortical capsular infarct or the neurobiological mystery of subsequent motor recovery, which bring about a critical gap in the basic science research of stroke. One of the significant reasons is that there is no proper animal model for subcortical capsular infarct (Sozmen et al., 2009).

Internal capsule, a crucial anatomical structure, contains corticospinal tract (CST), which is responsible for transmitting motor signals from multiple motor cortices to the inferior motor neurons of spinal cord. Meanwhile, most of the corticospinal fibers pass through the posterior part of the posterior limb of internal capsule (PLIC). However, the lesion of PLIC in animal models of subcortical white matter ischemia research has been limited. The rodent brain has substantially less white matter than humans and primates, which is a principal problem in modeling white matter stroke in it (Sozmen et al., 2009). In addition, the irregular and narrow internal capsule of a rat makes it hard to access and control the extent of lesion (Kim et al., 2014).

The clinical features and recovery patterns of capsular infarct are various, mainly depending on the location and size of internal capsule infarct lesion. Besides, the mechanisms of cell repair and death are likely to be different in capsular infarct caused by the occlusion of terminal arteries and subcortical stroke from large artery gray matter strokes, which leads to different ways of sensorimotor recovery (Shannon et al., 2007). Previous studies demonstrated that if the capsular infarct lesion extends into the PLIC or the thalamus, the prognosis is poor as the damage to the efferent fibers of the CST would diminish the possibility of compensation (Fries et al., 1993). Only a few models successfully showed persistent and obvious sensorimotor deficits after subcortical capsular infarct (Lecrux et al., 2008; Sozmen et al., 2009). The detailed study on correlation between the location and extent of capsular infarct and its clinical manifestation and recovery patterns has not been reported yet.

Recently, cerebral plasticity of the surviving neurons has been investigated on compensating for the functional defect after stroke. Neuroimaging, such as functional magnetic resonance imaging (fMRI), has been extensively used to understand the mechanism of brain function during stroke recovery (Mohanty et al., 2018; Shim et al., 2016). Evidence from neuroimaging studies on the striato‐capsular stroke indicated that the recruitment of supplementary motor structures and the enhanced activation of bilateral motor pathways could be detected after stroke (Gerloff et al., 2006; Volz et al., 2015). Some neuroimaging studies reported that the integrity of CST is important for predicting clinical outcomes and evaluating the sensorimotor recovery potential in capsular infarct (Lindenberg et al., 2010; Ward et al., 2006). However, there are few neuroimaging studies in the model of pure circumscribed capsular infarct in PLIC.

In the present study, we established a rat model by using a photothrombotic technique to make the circumscribed capsular infarct lesioning (Kim et al., 2015). The fMRI was used to investigate the brain activity under the passive forearm movement.

## 2 MATERIALS AND METHODS

Experiments were conducted on 36 rats with weights ranging from 250 to 300 g. All animal use procedures were strictly in accordance with the guide for the care and use of laboratory animals.

### 2.1 Animal groups

Control normals: 18 normal rats

Experimental group: 18 capsular infarct rats

### 2.2 Capsular infarct models

The method of establishing the photothrombotic capsular infarct model was as follows: (1) The experimental animals were induced with 5% isoflurane in the anesthesia box for about 2 min and then anesthetized with 2%–2.5% isoflurane; (2) heads of rats were fixed with a small animal stereotactic frame; (3) a blanket was placed under the experimental rats; (4) rectal temperature was maintained at about 37.5°C; (5) the heads of rats were shaved and sterilized; (6) a small cranial window was made on 2 mm posterior to bregma and 3.1 mm lateral to midline with animal skull drill. In the process of craniotomy, ice normal saline was used to reduce the temperature of skull drill and skull surface to reduce the damage to cerebral cortex; (7) right PLIC was precisely positioned by stereotactic device; (8) rose bengal dye with 20 mg/kg was injected through tail vein; (9) a thin optical fiber with a diameter of 105 μm was slowly placed to reach the PLIC with the help of casing pipe, and a 520 nm laser transmitter was connected to the other end of the fiber (intensity of approximately 4.0 mW) and then irradiated with a laser for 1.5 m; (10) the local wounds were sutured and disinfected; the rats were released from the stereotactic frame and transferred to the cage after they woke up.

### 2.3 Behavioral assessment

Before operation and on the first and seventh day after operation, the modified Ashworth score was performed by technicians who did not know the grouping situation (Min et al., 2012). Modified Ashworth is the main clinical measure for evaluating muscle spasms in neurological diseases and is a six‐grade scale. The scores range from 0 to 4 with 0 representing normal muscle tone. A higher score indicates an increase in spasticity or passive resistance to movement.

### 2.4 MRI acquisition (imaging)

All the animals underwent fMRI 1 week after the modeling process. All scanning was performed with a Bruker BioSpec 70/30USR 7T MRI scanner (Bruker, Billerica, MA) at a comprehensive university. A Bruker inner diameter (85 mm transmit‐only volume coil) in combination with an anatomically shaped Bruker mouse brain four‐channel phased array receive‐only surface coil (10–20 mm internal diameter; Bruker, Billerica, MA) was used. To improve the homogeneity of the B0 field, a Bruker fast map shimming program was performed. High‐resolution anatomical images were acquired with a fast‐spin echo sequence (RARE; T2 weighted study) for 256 s. Excitation pulse was 90 degrees. The parameters that we used are as follows: a field of view of 20 mm × 20 mm, an in‐plane resolution of 256 voxels × 256 voxels, spatial resolution of 0.078 mm × 0.078 mm, gap of 0.1 mm, slice thickness of 0.1 mm, 167 slices, repetition time = 3000 ms, and echo time = 35 ms. Used echo planar imaging (EPI) sequence was a gradient echo. The following EPI parameters were used: spatial resolution of 0.313 mm × 0.313 mm, a field of view of 20 mm × 20 mm, slice thickness of 0.3 mm, 32 slices, and an in‐plane resolution of 64 voxels × 64 voxels. EPI images were acquired with repetition time = 1000 ms, echo time = 37.323 ms, and 600 volumes per animal.

During scanning, the rat was anesthetized with halothane gas under an induction dose of 5% and a maintenance dose of 1.5%. Before the MRI scan, two MRI‐compatible electrodes were inserted into the second and third digits and the fourth and fifth digits of the left forepaw of the rat and connected to a constant current stimulator (2.0 mA, 1 Hz) synchronized with the MRI scan.

### 2.5 Imaging preprocessing

All steps were carried out in the Statistical Parametric Mapping (SPM8) (http://www.fil.ion.ucl.ac.uk/spm), on a MATLAB 2013b platform (The MathWorks Inc., https://www.mathworks.com). Preprocessing steps included (1) brain extraction, (2) band‐pass filtering (0.01–0.1 Hz), (3) slice timing correction, (4) spatial smoothing (a Gaussian kernel FWHM of 0.7 mm), and (5) normalization to standard space. Then the first and second levels of fMRI statistical analysis were performed for determining the brain activation area.

### 2.6 Statistical analyses

Independent sample *t* tests were performed to assess differences between data from experimental group and control normals (*p* < .001). In addition, AlphaSim estimation was used for multiple comparisons at *p* < .05.

## 3 RESULTS

### 3.1 Results of modified Ashworth score and neurohistologic findings

The preoperative scores of all rats were 0. On the first day after operation, the scores of the experimental animals were 1–3. On the seventh day after operation, the muscle tension was still increased, and the scores were 1–2 (see Figure S1). The histological examination of specimens taken 1 week after establishing the photothrombotic capsular infarct model revealed well‐defined infarct lesions in the PLIC. When viewed under low‐power magnification, a circular‐to‐ovoid cystic cavity was observed. The mean infarct volume, which includes the area of necrosis and surrounding demyelination, was measured to be 0.50 ± 0.21 mm^3^ across serial sections. There was no significant correlation between the change in the modified Ashworth score and the infarct volume.

### 3.2 The brain activity comparison under left forearm passive movement in controls

**FIGURE 1:**
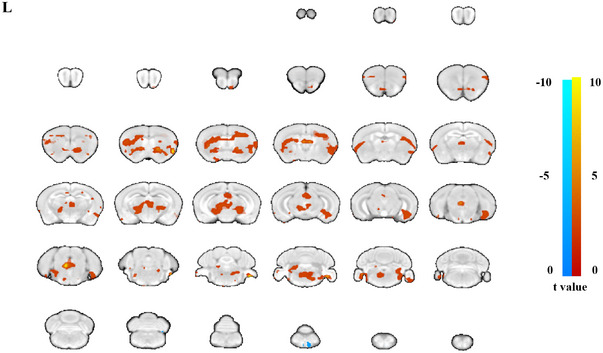
The brain activity comparison under left forearm passive movement in controls. Significant brain activation enhancement were found in caudate putamen frontal association somatosensory cortex, thalamus dorsolateral, and thalamus midline dorsal.

All the animals underwent fMRI 1 week after the modeling process. We found significant brain activation enhancement in caudate putamen frontal association somatosensory cortex, thalamus dorsolateral, and thalamus midline dorsal (Figure 1 and Table 1).

**TABLE 1:** The brain activity in control group induced by left forearm passive movement.

Region	Coordinates			Voxels	*t* Value
	*x*	*y*	*z*		
caudate_putamen	−47	−11	−62	48	7.29
corpus_collosum	0	−7	−8	25	5.71
cortex_frontal_association	−52	24	−12	27	5.62
cortex_somatosensory	−27	0	3	164	4.43
thalamus_dorsolateral	−35	−10	−70	13	6.39
thalamus_midline_dorsal	−39	−6	−68	14	3.66

### 3.3 The brain activity comparison under left forearm passive movement in capsular infarct models

**FIGURE 2:**
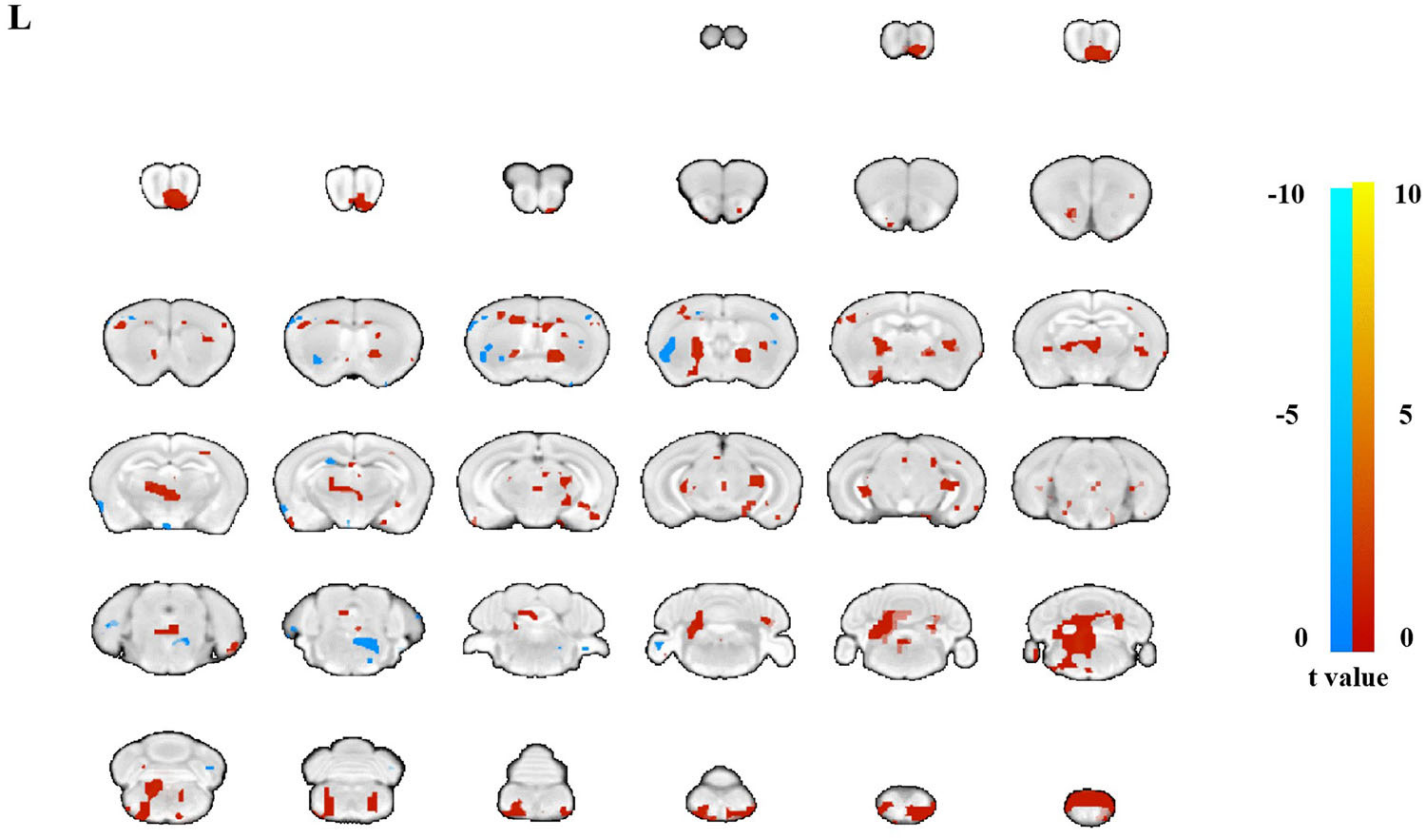
The brain activity comparison under left forearm passive movement in capsular infarct models. Less brain activation enhancement were found in somatosensory cortex, thalamus dorsolateral, and thalamus midline dorsal.

All the animals underwent fMRI 1 week after the modeling process. We found less brain activation enhancement in somatosensory cortex, thalamus dorsolateral, and thalamus midline dorsal (Figure 2 and Table 2).

**TABLE 2:** The brain activity in capsular infarct models induced by left forearm passive movement.

Region	Coordinates			Voxels	*t* Value
	*x*	*y*	*z*		
cortex_somatosensory	−20	20	15	27	3.23
thalamus_dorsolateral	−25	12	−27	12	4.23
thalamus_midline_dorsal	−2	18	−33	6	3.11

## 4 DISCUSSION

In this study, we analyzed the neural changes of brain activity in rats with circumscribed capsular infarcts. Our findings imply that PLIC is connected to these structures in function, interacts together with them, and, accordingly, the lesion of PLIC manifests the related symptoms.

Small animal stroke models can help us understand the pathophysiology and develop new therapeutic strategies for sensorimotor recovery. Cortical infarct models have been widely used in motor and somatosensory regions. In fact, cerebral artery occlusion is more likely to affect white matter in human patients. The degree of white matter damage affects the severity of sensorimotor impairment and the further clinical outcomes. Nonetheless, previous subcortical capsular stroke models with an insufficient degree of motor impairment and a rapid recovery process are difficult to meet the requirements of the comprehensive study of capsular infarct (Shibata et al., 2004; Tanaka et al., 2008). The capsular infarct model presented in this study shows the targeted destruction of the PLIC with marked and persistent subsequent motor impairment in forelimb functions. In this sense, this model simulates the human long‐term functional impairment induced by sensorimotor cortex lesions.

Photothrombotic capsular infarctions in rodents which is the selective destruction of the white matter without damaging the neighboring gray matter structures have been shown to successfully cause persistent motor deficits in the corresponding limbs, which has great merit over other techniques (Kim et al., 2014; Song et al., 2016). As the most critical white matter structure, the permanent damage of the CST in the PLIC is directly related to the subsequent severe motor dysfunction. There are extensive studies on the course and somatotopic distribution of the CST with diffusion tensor imaging techniques in humans, whereas similar studies on the anatomy of the CST in rodents are extremely rare. Therefore, the stereotactic coordinates used to generate capsular infarct models in the rat are different.

We observed that changes in the modified Ashworth score did not significantly correlate with infarct volume. This may be due to the fact that modifications in the Ashworth score are influenced not only by the local anatomical site but also by other factors, such as the increasingly accepted idea that various regions that cause similar symptoms may be linked within a common network in the brain (Fox, 2018). Thus, we should view functional impairments and recovery stemming from brain injury from the perspective of the overall functional circuit/network, rather than only focusing on the damaged local area. With the introduction of the concept of the “human connectome,” the developmental direction of functional imaging in clinical translation is being gradually realized. Accordingly, all the animals underwent fMRI 1 week after the modeling process. The fMRI, used to investigate the brain activity, after lesioning identified PLIC subregions by photothrombotic technique, is helpful to clarify this confusion and determine the appropriate target when generating new stroke models. In present study, our results showed the neural changes of brain activity after a circumscribed capsular infarct. In controls, the passive movement would induce strong activation in caudate, putamen, frontal association somatosensory cortex, thalamus dorsolateral, and thalamus midline dorsal; however, in capsular infarct models, the passive movement would only induce limited activation mostly in somatosensory cortex, thalamus dorsolateral, and thalamus midline dorsal. Capsular infarct could downregulate the cortical activity in sensory‐related structures, including capsular area and thalamus. Such findings imply that PLIC is connected to these structures in function, interacts together with them, and, accordingly, the lesion of PLIC manifests the related symptoms.

Our study delineated the relation between neural plasticity changes of brain activity detected by fMRI and circumscribed capsular infarct in PLIC using a photothrombotic technique. The fMRI has been proved that it can be used for determining cortical reorganization procedure in different states (Takenobu et al., 2014; Xu et al., 2014). The present study explored the neural plasticity of capsular stroke models at acute stage. Our results proved that capsular infarct induced decreases in brain activity in different cortical areas, although there were no cortical stroke lesions. These findings indicate the changes of intrinsic brain activity and dynamic signal transduction in neurovascular units and the temporal changes of local interaction in the process of neural plasticity after stroke. Further appropriate therapeutic interventions for repair potential are required to enhance in capsular infarct.

Despite its usefulness, our study has some limitations. First, this model does not reflect the full spectrum of human white matter stroke because photothrombotic destruction slightly differs from thromboembolism. Second, the results may be affected by the reorganization of functional connections after capsular stroke. Our study lacks a long‐term dynamic observation.

## 5 CONCLUSION

Such findings imply that PLIC is connected to these structures in function, interacts together with them, and, accordingly, the lesion of PLIC manifests the related symptoms. Further appropriate therapeutic interventions for repair potential are required to enhance in capsular infarct.

## AUTHOR CONTRIBUTIONS

Xu‐Yun Hua and Fei Teng designed the experiments. Xue‐Jia Li and Wen‐Xin Liu conducted animal experiments. Jie Ma analyzed data and wrote the manuscript. Xu‐Yun Hua and Fei Teng supervised the analysis of the data and help language editing. All authors read and approved the final manuscript.

## CONFLICT OF INTEREST STATEMENT

The authors declare that they have no competing of interests.

### PEER REVIEW

The peer review history for this article is available at https://publons.com/publon/10.1002/brb3.3125.

## Supporting information

Figure S1 Results of the modified Ashworth score. The preoperative scores of all rats were 0. On the first day after operation, the scores of the experimental animals were 1–3. On the seventh day after operation, the muscle tension still increased, and the scores were 1–2.

## Data Availability

The data that support the findings of this study are available from the corresponding author, upon reasonable request.
