# Supplementary figures and images for: Spatial patterns of intrinsic brain activity in rats with capsular stroke
Source: Brain Behav. 2023 Jul 6;13(8):e3125. doi: 10.1002/brb3.3125 (PMC10454278; doi:10.1002/brb3.3125)

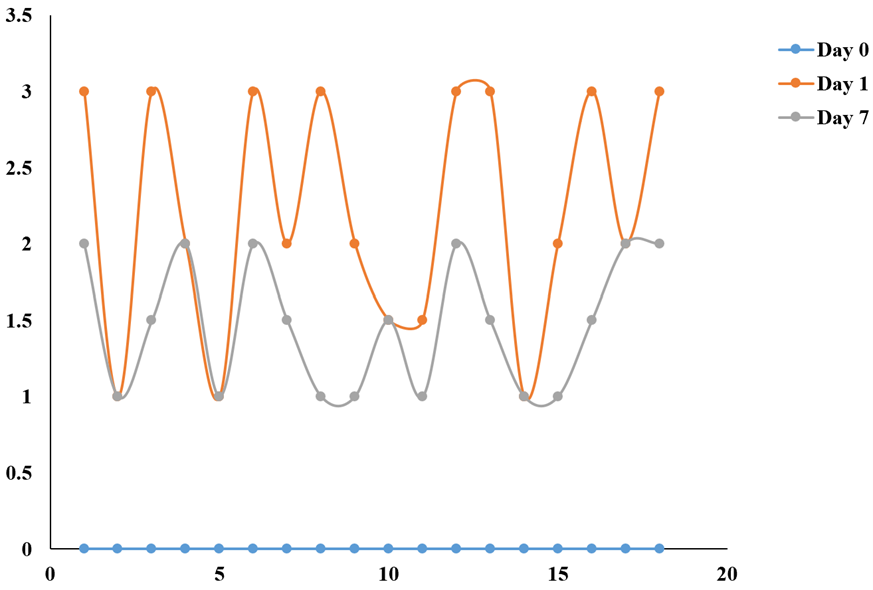

Supplement: Supplementary file 1 — Figure S1 Results of the modified Ashworth score. The preoperative scores of all rats were 0. On the first day after operation, the scores of the experimental animals were 1–3. On the seventh day after operation, the muscle tension still increased, and the scores were 1–2. [file BRB3-13-e3125-s001.tif]
